# A Strangulated Congenital Diaphragmatic Bochdalek Hernia Diagnosed in an Adult

**DOI:** 10.1155/2022/3355058

**Published:** 2022-07-20

**Authors:** Warsinggih Warsinggih, Julianus Aboyaman Uwuratuw, Arham Arsyad, Muhammad Faruk

**Affiliations:** ^1^Division of Digestive, Department of Surgery, Faculty of Medicine, Hasanuddin University, Makassar, Indonesia; ^2^Division of Digestive, Department of Surgery, Dr. Wahidin Sudirohusodo Hospital, Makassar, Indonesia; ^3^Department of Surgery, Faculty of Medicine, Hasanuddin University, Makassar, Indonesia

## Abstract

Although Bochdalek hernias are uncommon in adults and difficult to diagnose, such congenital diaphragmatic hernias (CDHs) are some of the most serious malformations among newborns. In particular, CDHs are accompanied by high mortality and marked disability later in life due to concomitant morbidity (i.e., caused by pulmonary lung hypoplasia and persisting pulmonary hypertension of neonates) and require long-lasting neonatal treatment in an intensive care unit. Late-diagnosed CDHs are extremely rare and always show small defects that justify a better prognosis than CDHs with large defects. In most cases, such CDHs go undiagnosed due to their mild, delayed symptoms. In the case described here, an 18-year-old Asian male presented with abdominal pain and a bowel contour in the epigastric region and left upper quadrant. A chest X-ray and thoracoabdominal computerized tomography scan confirmed the diagnosis of bowel obstruction due to a left diaphragmatic hernia. Moreover, a defect was revealed in the posterolateral left diaphragm, and the transverse colon, spleen, and half of the stomach had herniated through it. A segmentectomy was performed on the transverse colon, followed by an end-to-end anastomosis, a diaphragmatic plasty, and the placement of an intrapleural catheter. In conclusion, diagnosing a diaphragmatic hernia before surgery can be difficult due to its rarity and wide range of symptoms. Although prenatal diagnosis using ultrasonography is possible in up to 80% of fetuses, that method may not be available in all regions in Indonesia or in all countries, where doctors thus continue to face a diagnostic challenge when dealing with CDHs.

## 1. Introduction

Congenital diaphragmatic hernia (CDH) is defined by an abnormality in the diaphragm that causes the contents of the abdomen to protrude into the thoracic cavity, thereby impairing lung development [1, 2]. CDH is one of the most serious neonatal abnormalities and has a prevalence of one in every 3000 live newborns [3]. This type of hernia is found on the right side of the body in 15–20% of CDH cases and on the left side in 80–85% of cases. Occurrence on both sides of the diaphragm is rare [3, 4]. Approximately 55–67% of patients with CDH survive [5, 6].

CDH includes Bochdalek hernias (BHs) (70%), which are present in the posterior-lateral diaphragm, and Morgagni hernias (25–35%), which occur in the anterior or central diaphragm (2–5%) [6]. Because BH is common in newborns and toddlers and not in adults, diagnosing this type of hernia is difficult, especially because most patients are unaware of it due to the mild delayed symptoms of CDH [3].

We herein report an uncommon case in which an adult male was suffering from a left-sided strangulated CDH of Bochdalek's type, presenting with intermittent abdominal pain.

## 2. Case Presentation

An 18-year-old Asian male with a two-day history of sharp and intermittent pain on the left side of his abdomen presented to the emergency department (ED). The pain decreased while lying down and increased while eating. The patient was treated in the ED for three hours. Despite being treated with a strong opioid analgesic (fentanyl), the pain worsened, so he was referred to the surgery department for a consultation. The patient had a history of recurrent pneumonia as a toddler, and he reported experiencing tightness in the chest after eating over the previous 12-month period. This patient had no prior surgeries or trauma, did not drink alcohol, and smoked one pack of cigarettes daily.

**Figure 1:**
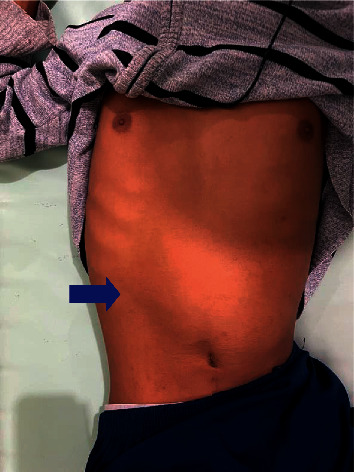
Physical examination showing bowel contour in the epigastric region and LUQ.

The patient complained of an inability to pass flatus or stool for 2 days. He was alert, and his vital signs were as follows: blood pressure of 100/70 mmHg; a temperature of 36.5°C; a respiratory rate of 22 breaths per minute; a heart rate of 108 beats per minute; and a Karnofsky score of 80. On auscultation of the right and left hemithorax, the patient's breath sounds were normal. The patient's abdominal examination showed a bowel contour in the epigastric region and left upper quadrant (LUQ) (Figure 1), and, on auscultation, bowel sounds were increased. An empty rectal vault was found after a digital rectal examination.

**Figure 2:**
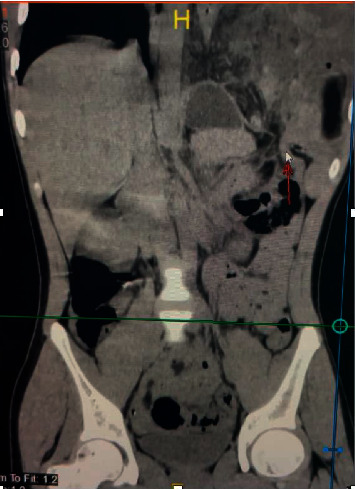
The thoracoabdominal CT scan without contrast showing a left-sided diaphragmatic defect (indicated by the white arrow) with herniation of the bowel into the thorax cavity (coronal view).

The left hemidiaphragm was elevated, and there was minimal pleural effusion evident in the posteroanterior view chest X-ray. The three-view abdominal radiograph series showed that the left hemidiaphragm was filled with gas, distended, and elevated. The thoracoabdominal computerized tomography (CT) scan without contrast showed a left-sided diaphragmatic defect with herniation of the bowel into the thoracic cavity (Figure 2). Laboratory findings showed slightly elevated leukocyte levels at 12.9 × 103/mL (normal values are 4.0–10.0 × 103/mL), and the results from blood gas analysis were within the normal range. Therefore, we diagnosed the patient with a bowel obstruction caused by a left diaphragmatic hernia.

**Figure 3:**
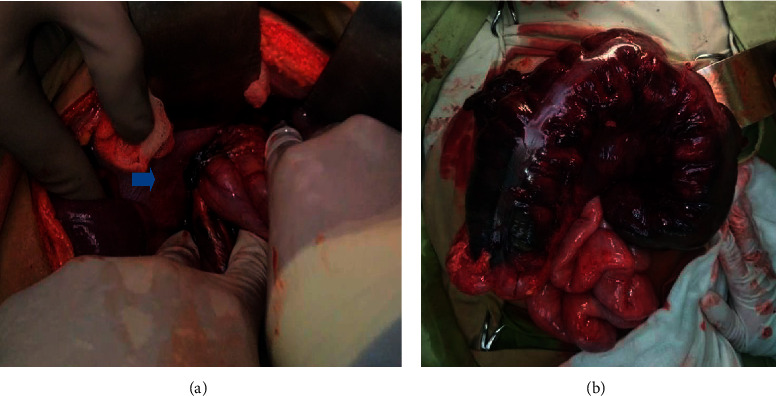
(a) The 8 cm defect in the posterolateral left diaphragm (indicated by the arrow), through which the colon transversum, spleen, and half of the stomach had herniated. (b) The colon transversum was completely necrotic.

After this, we performed an exploratory laparotomy via a midline incision, wherein the colon ascendens and colon transversum appeared extremely dilated. An obstruction was observed on the colon ascendens and a small part of the colon transversum. There was an 8 cm defect in the posterolateral left diaphragm, through which the colon transversum, spleen, and half of the stomach had herniated (Figure 3(a)). The colon transversum was completely necrotic (Figure 3(b)), and 200 ml of serohemorrhagic fluid drained from the thorax cavity. A segmentectomy on the colon transversum, an end-to-end anastomosis, and diaphragmatic plasty using a non-absorbable monofilament were performed, after which we inserted an intrapleural catheter. The left lung was fully inflated in the postoperative chest X-ray. The patient made a full recovery and was discharged 6 days later.

## 3. Discussion

Vincent Alexander Bochdalek was the first to describe BH in 1848 [7, 8]. A BH is a defect that occurs at the posterior aspect of the diaphragm [9]. Adults with BH are extremely rare, accounting for approximately 0.17–6.0% of all diaphragmatic hernias diagnosed. The defect is seen on the left side in 85% of BH cases, on the right side in roughly 10% of cases, and bilaterally in approximately 5% of cases [7, 8].

The etiology of CDH is mostly unknown, but it is currently assumed to be multifactorial. The majority of instances demonstrate a single diaphragmatic abnormality manifesting as pulmonary hypoplasia and persistent pulmonary hypertension in newborns. CDH is related to genitourinary, cardiac, and gastrointestinal abnormalities as well as chromosomal aneuploidy, such as trisomies [2]. A global collaborative effort found that a variety of possible genes and environmental factors play a role in the development of CDH in children. Despite being located on distinct chromosomes, the majority of the genes are known to be linked to the retinoic acid pathway. Interference with the enzyme retinaldehyde dehydrogenase 2 (RALDH2) at a vital moment in retinoic acid metabolism is a crucial step in the most widely used animal model of CDH (the nitrofen rodent model), particularly experimental models [10].

There are two common clinical presentations of adult CDH: (1) incidental detection during X-rays for symptoms unrelated to the diaphragmatic hernia or (2) symptoms that arise from a visceral rupture within the chest cavity, followed by organ strangulation and incarceration. Symptoms vary depending on which organ is affected: vomiting, dysphagia, and intermittent abdominal pain are common digestive symptoms, while chest pain and dyspnea are common respiratory symptoms [11]. Patients with a BH experience stomach pains more frequently, and patients with a Morgagni hernia experience these symptoms less frequently. Respiratory symptoms are significantly more frequent in patients with a Morgagni hernia. The stomach, small intestine, and spleen are the most commonly displaced organs in a BH, followed by the omentum, transverse colon, and descending colon [9]. In this case, the transverse colon, spleen, and half of the stomach had herniated, and the patient had intermittent abdominal pain.

A chest X-ray and barium assays were used to make the diagnosis. Organs, fluid, and air can be seen in the chest above the diaphragm [6]. The best technique for diagnosis is a CT scan, which provides the unique ability to assess the presence, location, and size of a defect and even allows examining the contents of distinct forms of diaphragmatic hernias. MRI is also beneficial but is frequently unavailable in an emergency [12].

Perrone et al. assert in a systematic review that surgery is the preferred treatment and that the preoperative context has a significant impact [6]. The surgical treatment for this disease is determined by the occurrence of visceral complications. Most studies suggest using the thoracic approach in an elective setting [11]. However, the abdominal approach is preferred in an emergency setting when a visceral abdominal lesion (including perforation of abdominal viscera, obstruction, malrotation, and strangulation) is difficult to identify and there are septic complications [11, 13]. We decided on the abdominal approach for this case because it was an emergency.

Due to recent advancements, minimally invasive techniques (thoracoscopy and laparoscopy) have become more feasible and safer, resulting in shorter hospital stays and decreased morbidity rates [6, 11, 14]. Brungardt et al. reported postoperative morbidity rates in patients undergoing CDH repair and concluded that outcomes are similar regardless of whether patients undergo an open or minimally invasive technique [15].

Compared to other congenital anomalies worldwide, the CDH mortality rate is high; the rates in Costa Rica and South America are 54.8% and 56.7%, respectively [16]. According to a recent study by the CDH Study Group, the size of the diaphragmatic defect is the most important factor impacting the prognosis of newborns with CDH, and it is also related to a greater mortality rate [17, 18]. The size of the defect is also the most likely predictor of the severity of the pulmonary hypoplasia [18] and the incidence of associated anomalies, such as neurodevelopmental delay [19], cerebral palsy [20], cardiovascular malformations, the number of abnormal organ systems, chromosomal aberrations, and frequent occurrence of liver in the chest [17].

In conclusion, definitive diagnosis of a diaphragmatic hernia before surgery is difficult because it is rare and has a variety of symptoms. X-rays and CT scans are helpful in detecting CDH. Prenatal diagnosis using ultrasonography is possible in up to 80% of fetuses, and that method may not be available in all regions in Indonesia or in all countries, where doctors thus continue to face a diagnostic challenge when dealing with CDHs.

## Data Availability

The data used to support the findings of this study are available from the corresponding author upon request.
